# Supplementary material for: COVID-19 Survivor Patients Carrying the Rs35705950 Risk Allele in MUC5B Have Higher Plasma Levels of Mucin 5B
Source: Curr Issues Mol Biol. 2022 Jul 22;44(8):3283–90. doi: 10.3390/cimb44080226 (PMC9329856; doi:10.3390/cimb44080226)
Supplement: Supplementary file 1 [file cimb-44-00226-s001.zip › cimb-1804639-supplementary.pdf]

Table S1. Genotype and allele frequencies of *SERPINA1* and *MUC5B* polymorphisms in patients included in the present study.

Genotype/ allele	IMV n=945 (%)	non-IMV n=361 (%)	p-value	OR	CI, 95%
**rs17580**					
TT	95.42	97.51		1 (Reference)	
AT	4.47	2.49	0.079	1.83	0.88-3.80
AA	0.11	0			
T	97.66	98.75			
A	2.34	1.25	0.106	1.89	0.92-3.90
**rs28929474**					
CC	99.32	99.17		1 (Reference)	
CT	0.55	0.83	0.877	0.63	0.15-2.66
TT	0.13	0			
C	99.74	99.58			
T	0.26	0.42	0.819	0.63	0.15-2.66
**rs35705950**					
GG	93.12	93.33		1 (Reference)	
GT	6.77	6.11	0.884	0.19	0.01-2.11
TT	0.11	0.56			
G	96.51	96.39			
T	3.49	3.61	0.977	0.96	0.60-1.53

IMV: patients requiring invasive mechanical ventilation; non-IMV: patients without invasive mechanical ventilation.

Table S2. Genotype and allele frequencies of *SERPINA1* and *MUC5B* polymorphisms in patients with COVID-19 and ARDS classified by PaO_2/FiO_2.

Genotype/ alleles	Mild n=280 (%)	Moderate n=641 (%)	Severe n=293 (%)	p-value	OR	CI, 95%
**rs17580**						
TT	95.79	95.79	96.49		1 (Reference)	
AT	4.21	4.06	3.38	0.921	0.79	0.33-1.90
AA	0	0.15	0.13			
T	97.89	95.94	98.31	0.662		
A	2.11	4.56	1.69		0.79	0.33-1.89
**rs28929474**						
CC	98.74	99.38	99.66		1 (Reference)	
CT	1.13	0.62	0.34	0.270	0.29	0.03-2.85
TT	0.13	0	0			
C	99.44	99.69	99.68	0.346		
T	0.56	0.31	0.32		0.29	0.03-2.86
**rs35705950**						
GG	93.23	93.32	92.88		1 (Reference)	
GT	6.02	7.52	7.12	0.862	1.18	0.60-2.32
TT	0.75	0.16	0			
G	96.24	96.58	96.44	0.874		
T	3.76	3.42	3.56		0.94	0.50-1.76

Mild: patients with mild COVID-19. Moderate: patients with moderate COVID-19, Severe: patients with severe COVID-19. p-value, OR and CI showed were between severe and mild

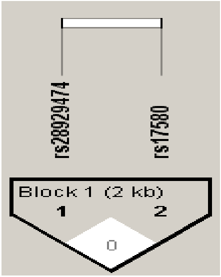

Supplementary Figure S1. The linkage disequilibrium analysis of rs17580 and rs28929474 of the *SERPINA1* gene in patients with COVID-19 survivors and non-survivors. We show r^2 (by: Haploview v.4.1)

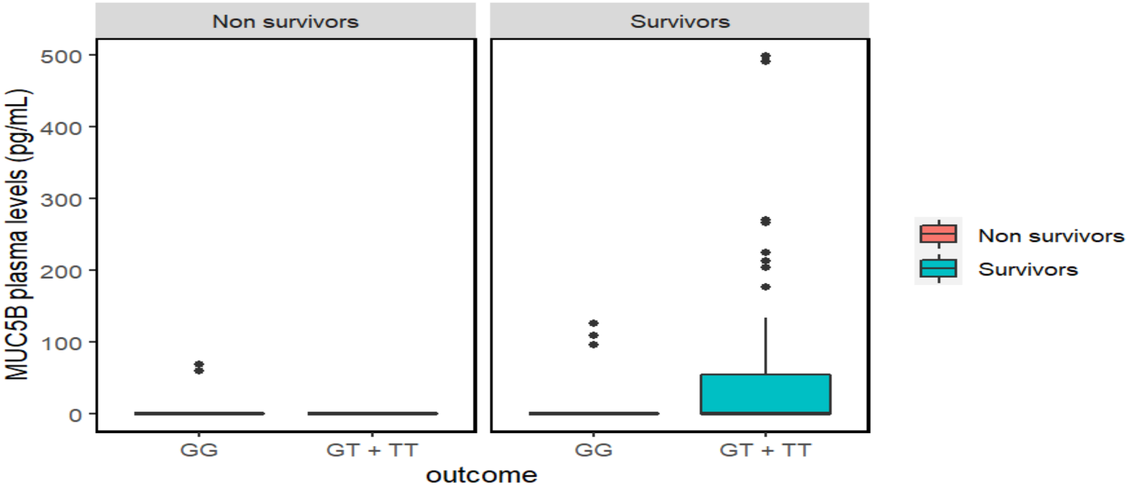

Supplementary Figure S2. Concentrations of mucin *5B* in plasma from patients with COVID-19.
